# Geographic and Research Center Origins of Rice Resistance to Asian Planthoppers and Leafhoppers: Implications for Rice Breeding and Gene Deployment

**DOI:** 10.3390/agronomy7040062

**Published:** 2017-09-21

**Authors:** Finbarr G. Horgan, Thanga Suja Srinivasan, Jagadish S. Bentur, Ram Kumar, K. Vasanta Bhanu, Preetinder Singh Sarao, Ho Van Chien, Maria Liberty P. Almazan, Carmencita C. Bernal, Angelee Fame Ramal, Jedeliza B. Ferrater, Shou-Horng Huang

**Affiliations:** 1 School of Life Sciences, University of Technology Sydney, 15 Broadway, Ultimo, Sydney, NSW 2007, Australia; 2 Centre for Plant Molecular Biology and Biotechnology, Tamil Nadu Agricultural University, Coimbatore, TN 641 003, India; sujasree07@gmail.com (T.S.S.); shhuang@dns.caes.gov.tw (S.-H.H.); 3 International Rice Research Institute, DAPO Box 7777, 1301 Metro Manila, Philippines; m.l.almazan@irri.org (M.L.P.A.); c.bernal@irri.org (C.C.B.); jferrater@gmail.com or jedeliza.ferrater@eastwestseed.com (J.B.F.); 4 Directorate of Rice Research, Rajendrangar, Hyderabad, AP 500 030, India; jbentur@yahoo.com; 5 Pioneer Hi-Bred Private Limited, 3rd Floor Babukhan’s Millennium Centre, 6-3-1099/1100, Raj Bhavan Road, Somajiguda, Hyderabad, AP 500 082, India; Ram.Kumar@pioneer.com; 6 Andhra Pradesh Rice Research Institute and Regional Agricultural Research Station, Maruteru, AP 534 122, India; vasanta99@yahoo.com; 7 Plant Breeding and Genetics Department, Punjab Agricultural University, Ludhiana, PB 141 004, India; preetento@gmail.com; 8 Southern Regional Plant Protection Centre, Ling Dinh 860000, Vietnam; hvchien@vnn.vn; 9 School of Environmental Science and Management, University of the Philippines, Los Baños, 4030 Laguna, Philippines; angelee.ramal@gmail.com; 10 Laboratory of Entomology, Wageningen University and Research Centre, P.O. Box 8013, 6700 EH Wageningen, The Netherlands; 11 Chiayi Agricultural Experiment Station, Taiwan Agricultural Research Institute, No. 2, Minquan Rd., Chiayi 60044, Taiwan

**Keywords:** brown planthopper, genotyping, green leafhopper, phenotyping, single-nucleotide polymorphism, SSST, virulence adaptation, whitebacked planthopper

## Abstract

This study examines aspects of virulence to resistant rice varieties among planthoppers and leafhoppers. Using a series of resistant varieties, brown planthopper, *Nilaparvata lugens*, virulence was assessed in seedlings and early-tillering plants at seven research centers in South and East Asia. Virulence of the whitebacked planthopper, *Sogatella furcifera*, in Taiwan and the Philippines was also assessed. Phylogenetic analysis of the varieties using single-nucleotide polymorphisms (SNPs) indicated a clade of highly resistant varieties from South Asia with two further South Asian clades of moderate resistance. Greenhouse bioassays indicated that planthoppers can develop virulence against multiple resistance genes including genes introgressed from wild rice species. *Nilaparvata lugens* populations from Punjab (India) and the Mekong Delta (Vietnam) were highly virulent to a range of key resistance donors irrespective of variety origin. *Sogatella furcifera* populations were less virulent to donors than *N. lugens*; however, several genes for resistance to *S. furcifera* are now ineffective in East Asia. A clade of International Rice Research Institute (IRRI)-bred varieties and breeding lines, without identified leafhopper-resistance genes, were highly resistant to the green leafhopper, *Nephotettix virescens*. Routine phenotyping during breeding programs likely maintains high levels of quantitative resistance to leafhoppers. We discuss these results in the light of breeding and deploying resistant rice in Asia.

## 1 Introduction

Host plant resistance (a plant’s ability to reduce herbivore fitness and deter damage) is regarded as an effective method to reduce the damage caused by herbivores and diseases to crop plants [1–5]. Rice, *Oryza sativa* L., resistance to planthoppers (Delphacidae) and leafhoppers (Cicadellidae) is one of the best-studied cases of host plant resistance for crop protection [6–8]. Rice is attacked by a range of planthoppers and leafhoppers. Some of these, e.g., the brown planthopper, *Nilaparvata lugens* (Stål), whitebacked planthopper, *Sogatella furcifera* (Horváth), and green leafhopper, *Nephotettix virescens* (Distant), severely damage rice either directly through feeding or by transmitting rice viruses [7,9]. During the 1970s and 1980s, the International Rice Research Institute (IRRI) released a series of high-yielding rice varieties with resistance to *N. lugens* and *N. virescens*. These varieties, many of which contained the *Bph1* or *bph2* genes for resistance to *N. lugens*, were planted throughout Asia [6]; however, the utility of these genes was often short-lived as adapted planthopper populations emerged soon after the varieties were first adopted by farmers [7,10,11]. In response to planthopper adaptation to *Bph1* and *bph2* resistance, current breeding programs have sought to release new varieties with resistance based on novel (not widely deployed) genes, including *Bph3/Bph32* (from Rathu Heenati or PTB33), *bph4* (from Babawee), and *Bph14* and/or *Bph15* (introgressed from *Oryza officinalis* Well ex Watt) [12–15]. Breeders have also sought to increase the strength and durability of resistance by pyramiding genes (introgressing two or more resistance loci into a single rice line) using marker-assisted selection [7,16].

The case of planthopper adaptation to varieties with the *Bph1* and/or *bph2* genes highlights the complexity of achieving lasting resistance in crop plants. Resistance is the result of a combination of genetically based plant traits that are often expressed during different plant stages under the influence of environmental factors, including crop management [6,17,18]. Furthermore, the success of host plant resistance depends on the extent of co-evolution between the target herbivore and varieties with major resistance genes, as well as the insect’s previous experience with specific host phenotypes and their quantitative resistance [19–22]. Although research has mainly focused on identifying and transferring major resistance loci to modern varieties, it is also apparent that planthoppers may experience declines in fitness for several generations when moving between two susceptible rice hosts without any major resistance genes [20,22,23]. This suggests that the strength and durability of resistance may be enhanced by combining quantitative resistance traits (usually identified as quantitative trait loci (QTLs) with major resistance (qualitative) genes [23,24]. Quantitative resistance traits are still poorly understood, but are likely related to biochemical or anatomical features of the host, including volatiles or surface chemicals that determine herbivore preferences and feeding responses [25]. These represent a barrier for planthoppers switching between natal (where the planthoppers developed) and new hosts, particularly where the two varieties are genetically distant [19,20]. Standard phenotyping methods, such as seedbox seedling tests that are commonly used in rice breeding programs will often function by promoting quantitative resistance during routine varietal screening [5,26,27], although they also verify the presence of major genes in resistance-breeding programs [11,16,25].

In the present study, we systematically examine resistance to *N. lugens* among a range of rice varieties that has been used in modern rice breeding programs and for which much of the underlying genetics of resistance has been determined. Because breeding programs tend to focus on single genes or single donor varieties [7,12–14], comparative studies with multiple donor varieties using a range of planthopper populations are rare [11,16,22]. Furthermore, breeding programs have focused predominantly on resistance in rice seedlings with little information available on resistance in older plants [7,16]. We therefore examined the responses of East Asian and Indian planthopper populations to a large collection of donor varieties using two standard tests that provide information on resistance or tolerance (the plant’s ability to compensate for damage) in seedlings and in older, tillering plants. We conducted a phylogenetic analysis of the varieties using single-nucleotide polymorphisms (SNPs) to assess the relation between accession origin (region or research center), genetic distance, and current virulence for these highly resistant varieties. Based on the common geographical origins and co-evolutionary history between South Asian planthopper populations and several resistant varieties, we hypothesized that East Asian populations would be less virulent to resistance genes and donor varieties of South Asian origin and conversely that Indian planthopper populations will show relatively higher levels of adaptation to South Asian resistance genes. Because many of the most resistant varieties are known to contain ≥2 major resistance genes, our experiments also allowed us to assess the potential for planthopper populations to adapt to future pyramided resistant lines (i.e., the probability that individuals virulent to varieties with complex resistance occur in natural populations). Using the same plant materials, we further examined the current status of resistance to *S. furcifera* at two sites in East Asia and examined resistance to *N. virescens* at one site in the Philippines. Because the relative contributions of plant resistance and plant tolerance in reducing herbivore damage are difficult to determine based on seedbox screening methods, we also conducted a series of fitness bioassays to assess the major categories of plant–herbivore interaction for the most resistant plant materials. To our knowledge, this is the largest systematic study of resistance and regional virulence in plant- and leafhoppers. The results of this study are discussed in light of improving strategies for the breeding and deployment of resistant rice varieties in Asia.

## 2 Materials and Methods

### 2.1 Herbivores

*Nilaparvata lugens* populations at seven rice research centers were included in the study. The colonies were initiated between 2004 and 2012 using wild caught individuals from rice fields located near each research center. The centers, with corresponding locations and years of planthopper collections, were as follows: (1) Directorate of Rice Research (DRR-India): (2010) Hyderabad, Andhra Pradesh, India; (2) Hi-Bred Private Ltd. (Pioneer-India): (2007) Medak, Andhra Pradesh, India; (3) Andhra Pradesh Rice Research Institute (APRRI-India): (2004) West Godavari, Andhra Pradesh, India; (4) Punjab Agricultural University (PAU-India): (2007) Ludhiana, Punjab, India; (5) Chiayi Agricultural Experiment Station (CAES-Taiwan): (2012) Chiayi, Taiwan; (6) Southern Regional Plant Protection Center (SRPPC-Vietnam): (2012) Ling Dinh, Vietnam; (7) International Rice Research Institute (IRRI-Philippines): (2009) Los Baños, Philippines.

We also evaluated resistance against *S. furcifera* colonies at two East Asian centers: CAES and IRRI. The colonies were initiated with wild-caught individuals collected during the same years and at the same locations as the corresponding *N. lugens* populations (indicated above). Resistance against a single *N. virescens* colony, located at IRRI, was also evaluated in the study. The colony was initiated with wild leafhoppers from rice fields in Laguna Province (Philippines) that were collected in 2008.

All colonies (*N. lugens, S. furcifera* and *N. virescens*) were initiated with ca. 500 adults placed on the susceptible variety Taichung Native 1 (TN1) (≥30 days after sowing) in wire mesh cages of 120 × 60 × 60 cm (H × W × L) under greenhouse conditions (temperatures ranged from 25 to 45 ^°^C, L12:D12 photoperiod). During the first two generations of rearing, the colonies were carefully monitored to eliminate diseased and virus carrying individuals.

### 2.2 Plant Materials

We used a collection of traditional rice varieties, landraces and modern varieties, as well as breeding-lines in our experiments. The collection was defined during a workshop in 2010 with rice breeders and entomologists from South and South East Asia. The original collection of 39 rice accessions was distributed to researchers in the region and has formed the basis for several comparative studies and rice breeding activities [11,16,22,28,29]. The varieties were selected to represent all the available *N. lugens* and *S. furcifera* resistance genes from the IRRI Genebank at the time of the workshop. Further details of the varieties used here and their putative resistance genes are presented in Table S1.

The varieties mainly represented the *O. sativa indica* subspecies; however, *O. sativa japonica* varieties (Asiminori and T65) were also included among the materials. Over 15 *N. lugens* resistance genes were identified from the materials we used, these were *Bph1, bph2, Bph3* (possibly including *Bph17* and/or *Bph32* [16]), *bph4, bph5, Bph6, bph8, Bph9, Bph10, Bph18, Bph20, Bph21, BPH25, BPH26*, and *Bph27*(t); Eight *S. furcifera* resistance genes were identified from the materials, these were *Wbph1, Wbph2, Wbph3, wbph4, Wbph5, Wbph6, WbphM1*, and *WbphM2*; the materials are also known to include the *Glh2* and *Glh9* genes against *N. virescens* and the *Zlh1* and *Zlh2* genes against the zig-zag leafhopper, *Recilia dorsalis* (Motschulsky). Furthermore, lines with notable resistance, such as Asiminori that expresses an induced ovicidal response (possibly with the *Ovc* gene: [30]) were included (Table S1).

Seed was acquired through the IRRI Genebank and from the Plant Breeding, Genetics and Biotechnology Division of IRRI. Only about 20 g of seed was available for most lines/varieties. Therefore, the seed was bulked-up in a screen-house at IRRI during 2010–2011 to attain enough seed for the experiments. The seed was shipped to centers outside the Philippines as 20 g packets following correct export–import protocols for each country. This ensured that all institutes used the same accessions and batches of seed in the experiments.

### 2.3 Genotyping

#### 2.3.1 Genotyping Assay

Pre-germinated seeds of 36 varieties were individually sown in size-10 pots (22 × 12 cm: H × R) filled with paddy soil and mixed with basal levels of ammonium phosphate fertilizer (0.8 g/pot). Seeds were covered with acetate cages (160 × 10 cm: H × R) and left to develop in a greenhouse at IRRI. Temperatures in the greenhouse fluctuated between 26–40 °C. The plants were watered regularly and weeded where necessary. When the plants were at the three to four leaf stage, samples of young leaves were collected into 2 mL micro tubes using liquid nitrogen (–196 °C) and stored at –20 °C for DNA extraction.

#### 2.3.2 DNA Extraction

A modified CTAB method adapted from Thomson [31] was used to extract DNA from the leaf samples. The young leaves were pulverized to a fine powder using liquid nitrogen and a micro pestle. 50 μg of the ground plant tissue was mixed with 750 μLof2× CTAB extraction buffer and 50 μL of 20% Sodium Dodecyl Sulfate (SDS). The suspension was mixed thoroughly and incubated at 65 °C in a water bath for 30–60 min with frequent agitation every 15 min. After incubation the suspension was cooled briefly and an equal volume of chloroform: isoamyl alcohol (24:1) was added. The solution was mixed thoroughly and centrifuged at 14,000 rpm for 15 min at 10 °C. After centrifugation, the upper aqueous phase was carefully transferred to a new 1.5 mL micro tube. An equal volume of isopropanol was added to the tube, mixed thoroughly and incubated at –20 °C overnight. After overnight incubation, the suspension was centrifuged at 14,000 rpm for 15 min at 4 °C. The supernatant was decanted and the DNA pellet was washed with 500 μL of 70% ethanol twice before being air dried. The pellet was then dissolved in TE buffer of 100 μL+1 μL of RNAse (Invitrogen™, Thermo Fisher Scientific, Carlsbad, CA, USA) (100 mg/mL) and incubated at 37 °C for 30 min in a heat-block. After incubation the DNA was precipitated using 10 μL of 3 M sodium acetate and 200 μL of absolute ethanol and incubated at –20 °C. The pellet was then dissolved in 50 μL of TE buffer and quantified using 0.8 percent agarose gel and a NanoDrop 2000 UV-Vis Spectrophotometer.

#### 2.3.3 Genotyping Using Infinium 6K Array

Whole genome genotyping of the samples was performed using the Illumina Infinium 6k array with 4606 single-nucleotide polymorphic (SNP) markers based on the Nipponbare rice genome. The array is based on two color fluorescent dyes, and allele calling of individual SNP markers is based on the intensity of dyes and clustering of three genotype groups by Illumina’s Genome Studio software [31].

### 2.4 Phenotyping for Planthopper Resistance

We used two different seedbox screening tests to evaluate the relative resistance/tolerance of the rice varieties to the *N. lugens* colonies at IRRI, SRPPC, CAES, DRR, Pioneer, APRRI and PAU. Because of poor germination of some rice lines, not all varieties were tested at each center. We used the same two methods to evaluate resistance against *S. furcifera* colonies at IRRI and CAES.

The standard seedbox screening test (SSST) is widely used in rice breeding programs throughout Asia and has been described in detail in several publications [25,32]. The modified seedbox screening test (MSST) was proposed by Velusamy et al. [32] to determine levels of resistance in older rice plants against damage from both nymphs and adult planthoppers. The MSST is proposed to better simulate field populations of planthoppers that consist of mixed generations with a slow density build-up. In contrast to the SSST, damage to plants in the MSST is caused by first generation nymphs as these develop to adults, and second generation nymphs emerging from eggs laid during the test. In the SSST, because of the higher density of nymphs and smaller size of the plants, plant mortality is quicker and the test is completed before nymphs reach the adult stage [32]. Comparisons of SSST and MSST results can demonstrate ontogenic shifts in rice resistance to planthoppers. The tests were conducted as follows:

#### 2.4.1 SSSTs

Seedboxes of 130 × 100 × 10 m (L × W × H) were filled with paddy soil to below the rim of the box. Twenty five to 30 seedlings of each rice variety were sown in lines from the edge of the box to before the middle of the box. The varieties were randomly assigned to rows in each seedbox with randomization conducted separately for each replicate. The susceptible check TN1 was interspersed among the test varieties as a central strip through the middle of the seedbox perpendicular to the rows, as extra rows at each end of the box, and at 5 row intervals interspersed with the test lines. A space of about 5 cm was left between adjacent seed rows. The seedlings were allowed to develop for 7 days after which time the rows were thinned to 20 plants per row. After 7 days, the seedlings were infested with newly emerged planthopper nymphs at a density of 8 nymphs per seedling. During the tests a mesh cage of 140 × 120 × 100 cm (L × W × H) was fitted neatly over each seedbox.

#### 2.4.2 MSSTs

The MSSTs were set up using the same seedbox dimensions as in the SSSTs; however, the seedlings were thinned to just 10 plants per row. When the plants were 20 days old, they were infested with planthopper nymphs at a density of 4 per seedling. After the plants were infested, a mesh cage (as above) was placed over each seedbox.

#### 2.4.3 Evaluation of Resistance

When the susceptible checks in the SSSTs or MSSTs were completely wilted due to planthopper feeding, the experiments were stopped and the condition of the seedlings scored using the standard evaluation system (SES) where 0 = no damage, 1 = slight damage to a few plants within a row, 3 = first and second leaves of each plant partially yellowing, 5 = pronounced yellowing or stunting of the plants or between 10% and 25% of plants wilted within a row, 7 = more than 50% of the plants wilted or dead and the remaining plants severely stunted or dying, and 9 = all plants wilted or dead.

### 2.5 Phenotyping for Leafhopper Resistance

We used a modification of the SSST to evaluate the varieties for resistance against the *N. virescens* colony at IRRI. Plastic bubble trays of 80 × 36 × 4cm(L × W × H) with 8 × 19 (rows × columns) each and 152 individual ‘bubble’ compartments were used. The trays were filled with paddy soil with no added fertilizer to below the rim of each compartment. Each rice variety was sown into four adjacent compartments with a space of about 4 cm between adjacent compartments in the trays. The different varieties were randomly assigned to positions in the bubble trays with randomization conducted separately for each replicate. The susceptible check TN1 was interspersed among the test varieties and also planted along the edges of the trays. The seedlings were allowed to develop for 7 days after which time they were thinned to 4 seedlings per compartment. On the day of infestation, the trays were individually covered with aluminum mesh cages of dimensions 90 × 48 × 82 cm (L × W × H). The seedlings were infested with newly emerged first-instar leafhopper nymphs at a density of 10 nymphs per seedling. When the susceptible check (TN1) was completely wilted due to insect feeding, the experiments were stopped and the condition of the seedlings was scored using the SES for rice as described above.

### 2.6 *Responses by* Nilaparvata lugens *to Resistant Varieties*

Standard seedbox tests have been criticized because they fail to differentiate between categories of plant–herbivore interaction (i.e., antibiotic-resistance, antixenotic-resistance, or tolerance). Furthermore, the most commony employed screening method, the SSST, focuses only on responses by plant- and leafhopper nymphs to relative levels of resistance among test varieties [2,5,25,32]. We conduced a series of fitness bioassays to clarify which interaction categories best explained the variations in damage to varieties in the standard seedbox tests. Using the IRRI *N. lugens* colony, we examined responses by planthoppers to 21 of the varieties known to possess genes or QTLs for resistance or tolerance to this planthopper species. Nymph survival and oviposition performance bioassays were carried out in a greenhouse at temperatures ranging from 25–45 °C. A biomass build-up bioassay was conducted in a screen-house facility with temperatures of 25–37 °C. Each bioassay was replicated 5–10 times in a completely randomized design. A further test, the honeydew production test, was conducted using the DRR planthopper colony. The test was conducted in a shaded screen house and replicated 5–6 times.

#### 2.6.1 Nymph Survival and Weight Gain

To determine the performance of nymphs on each rice variety, 10 newly emerged nymphs were placed together on 15-day old plants. Plants were produced from pre-germinated seedlings in clay pots (7 × 11 cm; H × D) each enclosed in a cylindrical acetate cage (61 × 0.5 cm; H × D) with a mesh side window and top for ventilation. After 15 days, the survivors were collected and oven-dried at 60 °C for 3 days.

#### 2.6.2 Oviposition

The number of eggs laid on each variety was determined by confining 2 gravid female planthoppers on 15-day-old plants for 3 days. Plants were produced from seedlings in clay pots (7 × 11 cm; H × D) each enclosed in a cylindrical acetate cage (61 × 10.5 cm; H × D) with a mesh side window and top for ventilation. After 3 days, the insects were removed and the plants were collected and frozen at –20 °C. These plants were later dissected and the number of eggs laid on each plant was counted under a stereomicroscope (10 × magnification).

#### 2.6.3 Biomass Build-Up

Two gravid female planthoppers were confined on 30-day-old plants in pots (22 × 24 cm; H × D). The rice plants (and insects) were enclosed in organza cages (150 × 22 cm; H × D). The organza cloth was fitted around a cylindrical acetate base cage (30 × 22 cm; H × D) stably embedded in the soil inside the pot and supported by bamboo stakes and aluminium wire rings. The top loose end of the cloth was tied to confine the insects. The females were left to lay eggs and the emerging nymphs were allowed to develop for 30 days. Planthoppers present in the cages after 30 days were collected using a mechanical aspirator and oven-dried during 7 days at 60 °C before being weighed.

#### 2.6.4 Honeydew Production

Ten day old seedlings of each variety were each infested with 2 gravid females in specially prepared, plastic feeding chambers. The feeding chambers confined the adults to within 5 cm of the base of the plants and were placed over filter paper treated with bromocresol green. After feeding, the area of excreted honeydew spots on the bromocresol-treated filter paper was measured using Image J software version 1.48 (National Institute of Health, Rockville, MD, USA).

### 2.7 Data Analyses

The genotype data acquired from the Genotyping Services Laboratory (IRRI) was saved as a tab delimited file in hapmap format with the .hmp extension. The file was imported to TASSEL 5.0 and the SNP markers were sorted using the sort genotype function. The markers with no allele calls were filtered with settings for a minimum count of 80% of the total samples and a minimum frequency of 0.01. The filtered file was saved as a .phy interleaved file. The .phy interleaved file was imported to MEGA6 where pairwise genetic distance was calculated using the Tamura and Nei model, with substitutions to include transition and transversion, and with a bootstrap value of 1000. All SNP marker positions with gaps and missing data were eliminated. The calculated pairwise genetic distances were used to construct a phylogenetic tree using the Unweighted Pair Group Method with Arithmetic Mean (UPGMA) in MEGA6 with a bootstrap value of 1000. The phylogenetic tree constructed in MEGA6 was saved as an .nwk file and imported to Figtree for further viewing and labeling of nodes and branches.

Resistance scores were compared for each test method (SSST or MSST) and planthopper population using general linear models (GLM) with scores ranked within tests. Pair-wise comparisons of damage scores against TN1 were conducted using Dunnett’s ‘many-to-one comparison’ tests. The appropriate Bonferroni adjustment was applied to α for the Dunnett’s tests. Results from the fitness bioassays were analyzed using GLM and compared using Pearson’s correlations. We graphically superimposed resistance results onto the phylogenetic tree to highlight clusters with high levels of resistance. We also included results from Horgan et al. [11] for *N. lugens* populations from South and South East Asia in the figure. The relations between damage scores and genetic distance (the distance of each variety from TN1) were examined using Spearman’s correlations for SSST and MSST scores from each center and for each herbivore species. To assess the utility of the donor varieties in terms of resistance strength and their potential to reduce damage from either *N. lugens* or *S. furcifera* in East Asia (Taiwan and the Philippines), we conducted multidimensional scaling (MDS) using Euclidean distances as proximity measures between varieties as calculated using a matrix of varieties (columns) and damage scores (rows). Multidimensional scaling detects patterns in a given proximity matrix which it represents as a simple geometric model. The MDS was conducted only for these two sites because data for *S. furcifera* resistance was not available from the remaining participating centers.

## 3 Results

### 3.1 Phylogenetics of the Rice Collection

**Figure 1:**
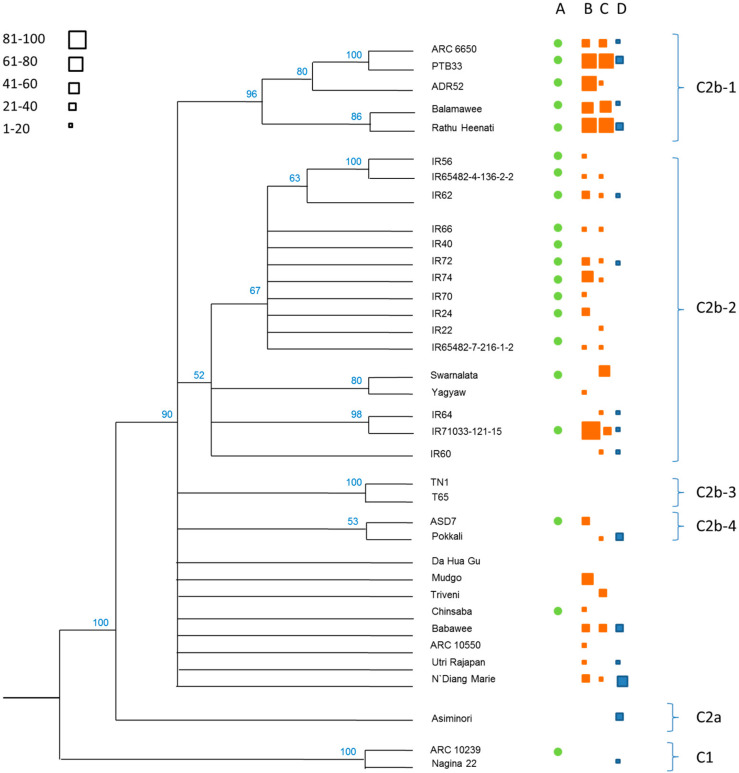
Phylogenetic relationships between 36 rice varieties indicating grouping into hierarchical clusters at six levels with related bootstrap values. Corresponding levels of resistance to planthoppers and leafhoppers as determined from this study and a related study (Horgan et al. [11]) are indicated as follows: (**A**) Varieties resistant to *Nephotettix virescens* (green circles, see also Figure 4); (**B**) varieties resistant to *Nilaparvata lugens* (orange squares, Horgan et al. [11]); (**C**) varieties resistant to *N. lugens* (orange squares, results from the present study, see also Figure 2); and (**D**) varieties resistant to *Sogatella furcifera* (blue squares, results from the present study, see also Table 1). The relative sizes of squares indicate standardized resistance ranking for each variety within each study (ranking from 1 (lowest) to 100 (highest)).

Genetic similarity analysis using 4606 SNP markers divided the varieties into two principal hierarchical clusters (Figure 1). We designate these as C1, containing only two of the Indian varieties—ARC 10239 and N22, and C2, which contained all other varieties. C2 was further divided between the Korean variety Asiminori alone (cluster C2a) and the remaining varieties (C2b). Hierarchical cluster C2b was further divided into 4 main clusters (C2b-1, C2b-2, C2b-3, and C2b-4). C2b-1 contained five highly resistant varieties of South Asian origin (India and Sri Lanka); C2b-2 mainly contained IR varieties and breeding lines, but also contained Swarnalata (Bangladesh) and Yagyaw (Vietnam); C2b-3 contained ASD7 and Pokkali (from South Asia); and C2b-4 contained the susceptible checks TN1 and T65 (both from Taiwan) as a clade with a bootstrap value of 100% (Figure 1). Several varieties within C2 appeared as unique varieties from relatively distinct lineages. These included varieties from Senegal, China, Indonesia, Myanmar, Sri Lanka and India that are recognized donors for the *Bph1, bph4, bph5, bph8, Wbph5* and *Wbph6* genes.

### 3.2 Virulence among Nilaparvata lugens Colonies

**Figure 2:**
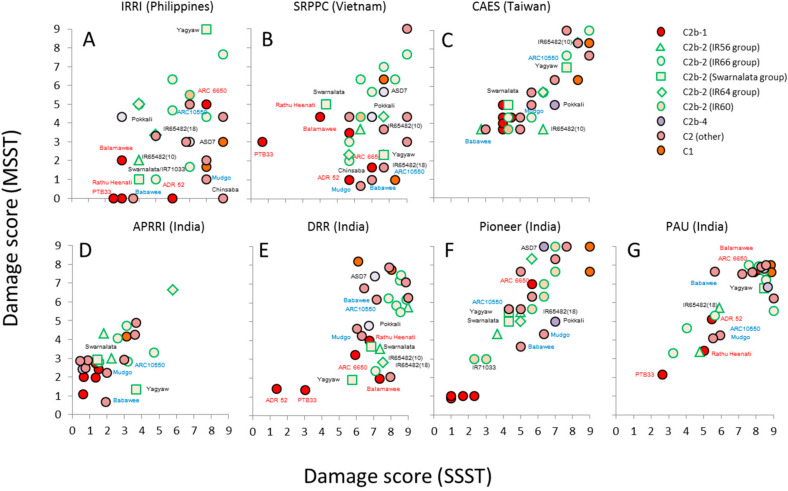
Bi-plots of damage scores from seedbox screening tests (SSSTs) and corresponding modified seedbox screening tests (MSSTs) for seven *Nilaparvata lugens* colonies. Key rice varieties are labeled (see also Table S2).

Figure 2 presents a series of bi-plots based on damage scores from the SSSTs and MSSTs for each of the seven planthopper colonies. In general, damage scores from the SSSTs were correlated with those from the MSSTs (Table S2), indicating that relative resistance was often stable between seedlings and older plants (Table S2). However, there was a tendency for varieties to perform better (lower damage scores) in the MSSTs than in the SSSTs conducted at IRRI, SRPPC and DRR, but performed better in the SSSTs at APRRI. High levels of resistance among the varieties from C2b-1 (see Figure 1) were noted across all centers; however, there were some notable exceptions: The IRRI and SRPPC populations were virulent to ADR52 seedlings. ARC6650 was often heavily damaged in both the SSSTs and MSSTs; furthermore, several tests conducted with colonies at SRPPC and PAU indicated high levels of damage to Balamawee and/or Rathu Heenati. These same two *N. lugens* colonies were also virulent against standard resistance donors such as Mudgo, ASD7, Babawee, and Chinsaba as well as rice lines with resistance genes introgressed from wild rice species. IR varieties thought to possess the *Bph1, bph2, Bph3/Bph32* and *bph4* genes performed poorly in tests with South East Asian populations (IRRI, SRPPC) and at DRR and Pioneer (with planthoppers collected from central Andhra Pradesh) (Figure 2). There was little relation between the genetic distance of each variety from TN1 and damage scores from either the SSSTs or MSSTs, except for the SRPPC results (Rho SSST = –0.445, *p* = 0.008; Rho MSST = –0.490, *p* = 0.003; all *p*-values for the remaining colonies and tests ≥ 0.068).

### 3.3 Virulence among Sogatella furcifera Colonies

‘Hopperburn’ and plant death were rare in tests with *S. furcifera*. Seventeen varieties showed resistance against *S. furcifera* from the CAES and IRRI colonies (Table 1). Damage scores from the SSSTs and MSSTs (Table 1) were highly correlated for both CAES (Rho = 0.704, N = 35, *p* < 0.001) and IRRI (Rho = 0.490,N=33, *p* = 0.004). Resistance was noted among varieties with the *Wbph1, Wbph2, wbph4*, and *Wbph5* genes and with *WbphN* and *WbphO* genes (MO1). Asiminori was also resistant to *S. furcifera*, particularly in the MSSTs (Table 1). Among the varieties with apparently high resistance to *S. furcifera* were PTB33, Rathu Heenati, Babawee, Balamawee and IR71033-121-15, each of which contains multiple resistance genes (albeit originally identified against *N. lugens*). The results suggest that *Wbph1, Wbph3, Wbph6, WbphAR, WbphM1*, and *WbphM2* were ineffective against the CAES colony, whereas *Wbph2, Wbph3, wbph4, Wbph6, WbphAR, WbphM1*, and *WbphM2* were ineffective against the IRRI colony. The Taiwanese colony appeared less virulent to the test varieties than the IRRI colony. Damage scores from the MSSTs at IRRI were correlated with the genetic distance of varieties from TN1 (Rho = –0.378, *p* = 0.025). All other correlations were non-significant.

**Table 1:** SSST and MSST damage scores for Chiayi Agricultural Experiment Station (CAES) and International Rice Research Institute (IRRI) *Sogatella furcifera* colonies; variety clades are indicated according to Figure 1.

Clade	Variety (Accession)	CAES (Taiwan)		IRRI (Philippines)	
		SSST^1^	MSST^1^	SSST^1^	MSST^1^
C1	ARC 10239	3.00 (0.00)^***^	3.67 (0.67)^**^	6.33 (1.00)	3.00 (0.00)
	Nagina 22	7.00 (1.15)	5.67 (0.67)	2.33 (1.00)^***^	1.00 (1.00)
C2b-1	ARC 6650	5.00 (2.00)	3.67 (0.67)^**^	5.00 (0.00)	3.00 (1.73)
	PTB33	4.33 (1.33)	3.00 (0.00)^***^	4.00 (1.00)	0.00 (0.00)^***^
	ADR52	5.00 (1.15)	4.33 (0.67)	3.00 (2.00)	1.00 (1.00)
	Balamawee	5.00 (1.15)	3.00 (0.00)^***^	3.00 (0.00)	1.00 (0.76)
	Rathu Heenati	5.00 (1.15)	3.00 (0.00)^***^	3.67 (0.00)	0.00 (0.00)^***^
C2b-2	IR56	-	-	5.67 (1.00)	3.00 (0.00)
	IR65482-4-136-2-2	4.33 (0.67)	4.33 (0.67)	3.00 (2.00)	2.00 (1.00)
	IR62	4.33 (0.67)	3.00 (0.00)^***^	4.33 (0.00)	2.00 (1.00)
	IR66	5.00 (0.00)	4.33 (0.67)	4.33 (1.00)	3.67 (0.67)
	IR40	8.33 (0.67)	7.00 (0.00)	4.33 (1.00)	3.67 (0.67)
	IR72	5.67 (0.67)	3.67 (0.67)^**^	5.00 (0.00)	3.00 (1.00)
	IR74	4.33 (0.67)	4.33 (0.67)	4.33 (1.00)	3.00 (0.00)
	IR70	5.67 (0.67)	6.33 (0.67)	4.33 (1.00)	3.67 (0.67)
	IR22	5.00 (0.00)	5.00 (0.00)	5.67 (1.00)	4.33 (1.33)
	IR24	5.00 (1.15)	4.33 (0.67)	5.00 (1.00)	3.00 (0.00)
	IR65482-7-216-1-2	4.33 (0.67)	5.67 (0.67)	4.33 (1.00)	3.00 (0.00)
	Swarnalata	5.00 (1.15)	4.33 (0.67)	4.33 (0.00)	3.00 (0.00)
	Yagyaw	7.00 (0.00)	6.33 (0.67)	5.00 (1.00)	3.00 (0.00)
	IR64	3.67 (0.67)	3.67 (0.67)^**^	5.00 (1.00)	2.00 (1.00)
	IR71033-121-15	4.33 (1.33)	3.67 (0.67)^**^	4.33 (1.00)	2.00 (1.00)
	IR60	4.33 (1.33)	3.67 (0.67)^**^	4.33 (0.00)	3.00 (0.00)
C2b-4	ASD7	7.00 (0.00)	5.00 (0.00)	5.67 (1.00)	1.00 (1.00)
	Pokkali	4.33 (1.33)	3.00 (0.00)^***^	2.33 (1.00)^***^	3.00 (0.00)
C2b	Da Hua Gu	7.67 (0.67)	7.00 (1.15)	5.00 (4.00)	3.00 (0.00)
	Mudgo	5.67 (0.67)	5.67 (0.67)	5.67 (3.00)	1.00 (1.00)
	Triveni	5.00 (1.15)	4.33 (0.67)	4.33 (1.00)	1.00 (1.00)
	Chinsaba	5.67 (0.67)	5.67 (0.67)	3.67 (1.00)	1.00 (1.00)
	Babawee	5.67 (0.67)	3.67 (0.67)^**^	3.00 (2.00)	0.00 (0.00)^***^
	ARC 10550	7.67 (0.67)	7.67 (0.67)	5.00 (2.00)	4.33 (2.96)
	Utri Rajapan	4.33 (0.67)	4.33 (0.67)	5.33 (0.00)	0.00 (0.00)^***^
	N’Diang Marie	5.00 (0.00)	3.00 (0.00)^***^	2.33 (1.00)^***^	0.00 (0.00)^***^
C2a	Asiminori	-	-	5.67 (1.00)	0.00 (0.00)^***^
-	ARC 11367	7.00 (1.15)	8.33 (0.67)	7.00 (0.00)	-
-	Jia Nong	66	-	-	-	2.00 (1.00)
-	MO1	3.00 (0.00)^***^	3.00 (0.00)^***^	3.00 (2.00)	-
C2b-3	T65	-	-	5.00 (1.00)	3.00 (0.00)
	TN1	8.33 (0.67)	7.67 (0.67)	7.17 (1.00)	5.33 (1.20)
	F-variety	3.059^***^	7.483^***^	1.921^**^	3.549^***^
	DF	34.00	34.00	37.00	36.00
	DF (error)	70.00	70.00	76.00	74.00

1 Comparisons with TN1 based on Dunnett’s test,

** *p* ≤ 0.01,

*** *p* ≤ 0.005. Standard errors are indicated in parentheses, N = 3; ‘-‘ indicates that the variety was not included in the corresponding test.

**Figure 3:**
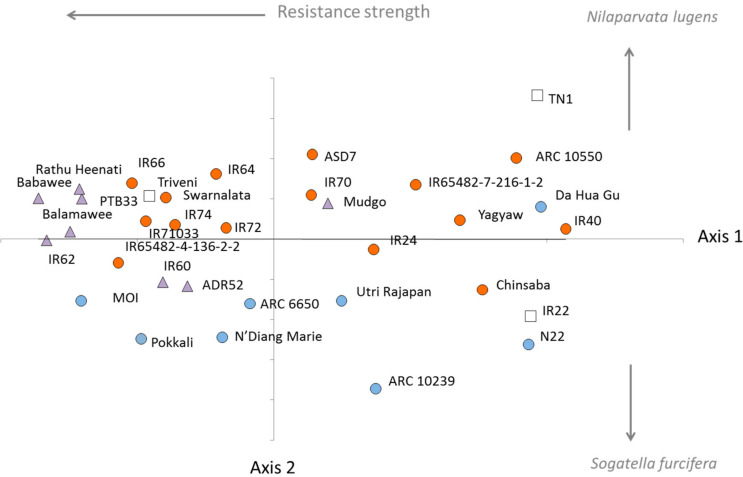
Multidimensional scaling plot based on Euclidean distances between resistance scores from SSSTs and MSSTs with CAES and IRRI *Nilaparvata lugens* and *Sogatella furcifera* colonies. Open squares indicate varieties with no known resistance, blue circles indicate varieties with known *S. furcifera* resistance genes, orange circles indicate varieties with known *N. lugens* resistance genes, and purple triangles indicate varieties with reported resistance against both planthopper species. Axis 1 represents the strength of resistance across all tests, planthopper species and colonies. Axis 2 represents the magnitude difference in resistance scores for *S. furcifera* and *N. lugens*. Stress = 0.02.

The multidimensional scaling plot (Figure 3) indicates that several varieties maintained relatively high resistance against *S. furcifera* and *N. lugens* in Taiwan and the Philippines (points left of axis 2 in Figure 3). Many of these varieties were resistant against both planthopper species albeit with stronger effects on *N. lugens* (indicated as points above axis 1 in Figure 3). The plot indicates that the *Bph1, bph2, bph5, bph8, Bph18, Wbph1, Wbph2, Wbph6, WbphM1* and *WbphM2* bestow only weak resistance in seedlings (SSST) and older plants (MSST) against planthoppers. In general, varieties with identified resistance genes against *N. lugens* were more effective against this planthopper species than against *S. furcifera*. However, the strong resistance of Pokkali against *S. furcifera* is apparent form the plot despite the variety having no identified *S. furcifera* resistance genes and the variety ADR52 appears more effective against *S. furcifera* than *N. lugens* possibly due to virulence adaptation to the *BPH25* and *BPH26* genes.

### 3.4 Response of Nilaparvata lugens to Resistant Rice

Egg laying, nymph biomass and population development (biomass) of *N. lugens* showed generally similar responses across the 21 resistant varieties examined at IRRI (Table 2). Varieties from clade C2b-1 with the the *Bph3/Bph32* and *Bph9* genes (PTB33, Balamawee and Rathu Heenati) demonstrated the highest levels of resistance. The line IR65482-4-136-2-2 (*Bph10*) and the traditional variety Babawee (*bph4*) had moderate levels of resistance. (Table 2). Nymph biomass, egg laying and population build-up were often highly correlated across the range of varieties and generally correlated well with the damage scores from the corresponding SSSTs and MSSTs (Table 3). The honeydew tests conducted at DRR indicated that feeding by *N. lugens* on most of the varieties was inefficient, except on Triveni. Honeydew production did not correlate with damage scores from the SSSTs at the same center (Pearson: C = 0.230, *p* = 0.316), but was correlated with scores from the MSSTs (C = 0.517, *p* = 0.020).

**Table 2:** Results from response bioassays with IRRI and Directorate of Rice Research (DRR) *Nilaparvata lugens* colonies on selected rice varieties; variety clades are indicated according to Figure 1.

Clade	Varieties	Nymph Biomass (mg)^1^,^3^	Number of Eggs per Plant^1^,^3^	Planthopper Biomass (mg) after 30 Days^1^,^3^	Honeydew Excretion (mm^2^)^2^,^3^
C2b-1	PTB33	1.08 (0.17)^***^	31.30 (7.16)	10.61 (2.79)^*^	10.67 (0.92)^***^
	Balamawee	0.69 (0.17)^***^	21.80 (3.26)^*^	17.97 (8.85)	13.67 (2.59)^***^
	Rathu Heenati	0.86 (0.16)^***^	24.60 (4.04)^*^	19.80 (2.10)	13.40 (2.87)^***^
C2b-2	IR65482-4-136-2-2	1.67 (0.17)	40.80 (10.50)	14.49 (5.48)^*^	-
	IR62	1.71 (0.18)	31.30 (6.65)	78.79 (35.39)	35.33 (3.24)^***^
	IR66	1.78 (0.13)	33.40 (4.32)	59.44 (19.44)	41.83 (16.18)^***^
	IR40	1.73 (0.16)	72.97 (9.99)	160.00 (40.00)	36.00 (12.95)^***^
	IR74	2.25 (0.10)	72.60 (10.80)	- 57.67 (11.04)^***^
	IR24	1.78 (0.16)	86.40 (7.57)	107.46 (27.76)	60.00 (5.52)^***^
	IR22	1.83 (0.25)	82.60 (10.23)	147.98 (20.23)	28.00 (4.34)^***^
	IR65482-7-216-1-2	2.46 (0.10)	68.00 (16.79)	147.98 (20.23)	33.80 (7.93)^***^
	Swarnalata	2.24 (0.15)	39.70 (9.17)	111.34 (24.53)	28.67 (8.01)^***^
	Yagyaw	2.05 (0.12)	53.70 (13.88)	98.08 (19.15)	21.50 (4.30)^***^
	IR64	2.52 (0.20)	76.20 (18.32)	77.73 (11.65)	38.80 (11.34)^***^
	IR60	-	-	-	28.60 (8.04)^***^
C2b-4	ASD7	2.15 (0.24)	63.70 (12.10)	52.51 (11.56)	-
	Pokkali	-	-	-	61.00 (5.13)^***^
C2b Mudgo	1.91 (0.30)	96.40 (16.85)	102.90 (22.58)	38.25 (5.02)^***^
	Triveni	1.52 (0.12)	82.20 (26.64)	93.10 (13.08)	123.25 (10.73)
	Chinsaba	2.01 (0.19)	53.00 (17.83)	21.54 (4.79)	17.20 (4.26)^***^
	Babawee	1.59 (0.21)^*^	37.90 (8.95)	17.14 (2.20)	39.67 (13.65)^***^
	Utri Rajapan	1.95 (0.17)	56.80 (12.57)	128.67 (23.46)	72.33 (18.51)^***^
C2b-3	TN1	2.40 (0.52)	81.80 (29.11)	107.57 (38.62)	133.00 (19.40)
	F-values	6.273^***^	3.007^***^	4.638^***^	8.266^***^
	Df	20	20	19	21
	Df (error)	174	174	80	93

1 Bioassay conducted with the IRRI *N. lugens* colony (N = 5–10);

2 Bioassay conducted with the DRR *N. lugens* colony (N = 5–6);

3 Comparisons with TN1 based on Dunnett’s test,

* *p ≤* 0.05,

*** *p ≤* 0.005. Standard errors are indicated in parentheses; ‘-‘ indicates that no test was conducted.

**Table 3:** Pearson correlation coefficients (above diagonal) and corresponding *p*-values (below diagonal) associated with *Nilaparvata lugens* (IRRI colony) resistance measures for a range of rice varieties.

Parameter	Egg Laying	Nymph (Biomass)	Population (Biomass)	SSST	MSST
Egg laying	-	0.579^1^	0.688^2^	0.662^1^	0.431^1^
Nymph (biomass)	0.006	-	0.555^2^	0.482^1^	0.389^1^
Population (biomass)	0.001	0.011	-	0.503^2^	0.587^2^
SSST	0.001	0.027	0.024	-	0.419^3^
MSST	0.051	0.082	0.007	0.014	-

1 N = 21;

2 N = 20;

3 N = 34.

### 3.5 Resistance against Nephotettix virescens

**Figure 4:**
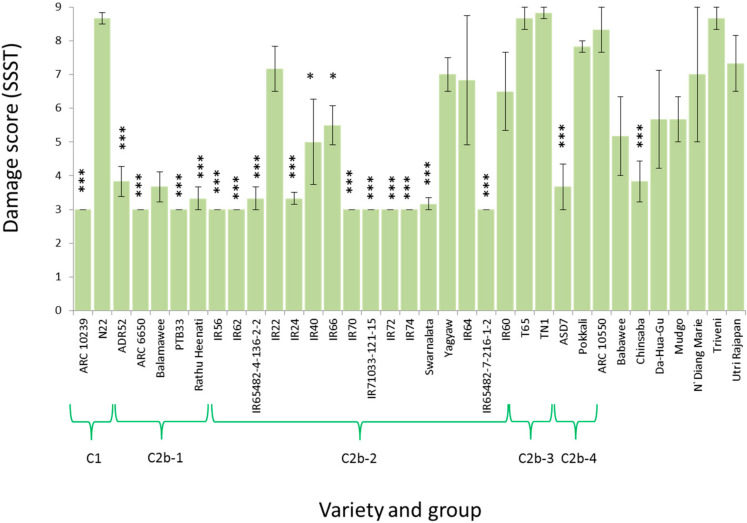
Damage scores from SSSTs with the IRRI *Nephotettix virescens* colony. Varieties that were significantly less damaged than TN1 are indicated with asterisks (Dunnett’s test, * *p* *≤* 0.05, *** *p* *≤* 0.005). Standard errors are indicated (N = 3). Variety clades are indicated according to Figure 1.

Varieties in clusters C2b-1 and C2b-2 had generally high resistance to *N. virescens* (Figure 4). Resistance to the leafhopper among varieties in C2b-1 (a clade with noted resistance to *N. lugens* and *S. furcifera*) indicates a broad spectrum of resistance (against three herbivore species) derived from the high number and diversity of major resistance genes among this group of traditional South Asian varieties. ASD7 with the *Glh2* gene, as well as ARC10239, Chinsaba, and Swarnalata (each without identified *N. virescens* resistance genes) were the only other traditional donor varieties with resistance against the leafhopper. It is noteworthy that most of the IR varieties with the exception of IR22 (an early IR variety with noted susceptibility to *N. virescens*), IR60 and IR64 were highly resistant to the leafhopper. Furthermore, within this cluster of relatively closely related lines, the non-IR line, Yagyaw, was susceptible to the leafhopper. There was no relation between genetic distance of varieties from TN1 and damage scores from the SSSTs for *N. virescens* (Rho = –0.0237, *p* = 0.177).

## 4 Discussion

Many of the varieties evaluated in the present study have been used in breeding programs either as donor varieties for resistance, as resistance checks in phenotyping studies (i.e., PTB33), or as sources of recognized resistance for the development of near-isogenic lines (NILs) for future rice breeding activities [7,10,16,17]. Furthermore, a considerable amount of information has been gathered on the levels or resistance to *N. lugens* and the nature of that resistance using the same accessions as evaluated here [11,29,30]. Despite this research attention, the materials had not been systematically evaluated for resistance against *S. furcifera* or *N. virescens* and, to our knowledge, no phylogenetic analysis of the materials had even been conducted. Our report on the phylogenetics of these varieties has revealed new information related to the potential origins of planthopper and leafhopper resistance. In particular, our results highlight that the most resistant rice varieties, which have been noted for their strong resistance in several previous studies [11,33,34], all belong to a single clade of Indian and Sri Lankan traditional varieties and landraces (cluster C2b-1 in Figure 1). However, a further Sri Lankan variety—Babawee—also with noted resistance from previous studies [13], did not form part of the high resistance clade and likely represents a second lineage of resistance sources (albeit with a single variety in our study). Furthermore, our results indicate that IR varieties (varieties developed at IRRI) were generally strongly resistant to *N. virescens* and included a cluster of closely related varieties (IR56, IR62 and IR65482-4-136-2-2) with relatively strong resistance against *N. lugens* in seedbox tests.

### 4.1 Aspects of Virulence Adaptation in Nilaparvata lugens

Currently some 85 genes for resistance to planthoppers and leafhoppers have been identified from rice and its wild relatives [7,14,17]. These genes continue to form a basis for rice resistance breeding programs in Asia; however, they are subject to planthopper and leafhopper adaptation. In recent years, virulent planthopper and leafhopper populations have been noted throughout Asia and these have rendered many major resistance genes ineffective [11,17,35,36]. For example, Horgan et al. [11] indicated that the *Bph1, bph2, bph5, bph7, bph8, Bph9, Bph10* and *Bph18* genes are currently ineffective against planthopper populations in many parts of Asia. This study expands on the study by Horgan et al. [11] by also examining resistance in older plants using the MSST.

Our phylogenetic analysis indicated that a single, closely related group of traditional rice varieties, the C2b-1 clade, included five of the most resistant rice varieties in our study, all of which originated in South Asia (India and Sri Lanka). However, virulence to these varieties, either in seedlings or as older plants, was apparent from a number of planthopper colonies. For example, the IRRI and SRPPC *N. lugens* colonies were virulent to ADR52 seedlings. ADR52 has been noted previously to have strong ontogenic changes in resistance. The *BPH25* and *BPH26* genes from ADR52 have been previously noted as ineffective in the Philippines and Vietnam [17]; however, according to our results, these genes likely play a role mainly in seedling resistance, with other, unidentified genes expressed in older plants. ARC6650 has been previously noted for its resistance to *S. furcifera* (Table S1); therefore it is unsurprising that the variety performed poorly against *N. lugens* at most centers; however, it remained largely undamaged in SSSTs and MSSTs at CAES and APRRI indicating that it does possess brown planthopper resistance albeit with reduced effectiveness because of current widespread virulence. It is significant to note that Balamawee and Rathu Heenati also performed poorly in tests in Vietnam (SRPPC), Punjab (PAU) and central Andhra Pradesh (DRR). Our comparative results indicate that *N. lugens* populations at PAU and SRPPC were highly virulent to several standard donor varieties: Swarnalata, Yagyaw, ASD7, Pokkali, Mudgo, Babawee and Chinsaba all performed poorly at PAU, and these varieties were often also heavily damaged by the planthopper population at SRPPC either in the SSSTs or MSSTs.

A number of studies have indicated that South Asian planthopper populations, especially those from the north of India and Bangladesh have been virulent to several resistance donors for some time [28,37–39]. Because the C2b-1 varieties each possess ≥2 resistance genes, our results indicate that individual planthoppers can be virulent to ≥2 genes and that planthopper populations can, at a minimum, have individuals each with virulence to different combinations of ≥2 resistance genes. It is also noteworthy that the Vietnamese *N. lugens* population had virulence to 4 of the 5 varieties in cluster C2b-1 in either the SSSTs or MSSTs, representing several resistance genes, as well as virulence to a number of further donor varieties as indicated above. This contrasts with historical evidence from the region that indicated low levels of virulence in the past [40,41], and suggests that the development of multiple virulence in Vietnam is a recent phenomenon [11,42]. Similarly, the IRRI planthopper population appeared virulent to several donor varieties, including some with multiple resistance genes (i.e., ADR52). Increasing levels of virulence have been noted among planthopper populations in the Philippines over the past decades [11,17,23,43].

A further feature of virulence adaptation revealed through the present study is the apparent capacity of planthoppers to gain virulence to rice lines with resistance gene loci introgressed from wild rice species. For example the lines IR65482-4-136-2-2 (*Bph10* from *O. australiensis*), IR65482-7-216-1-2-B (*Bph18* from *O. australiensis*) and IR71033-121-15 (*bph20, bph21* from *O. minuta*) often performed poorly, particularly when tested against East Asian populations. The first two of these lines also performed poorly in fitness tests conducted at IRRI (Table 2). The derived IR varieties that are thought to possess planthopper resistance genes through crossing with resistance donors (Mudgo, ASD7, PTB33, Rathu Heenati and Babawee) [10] also performed poorly in East Asia, particularly in the MSSTs at IRRI and the SSSTs at SRPPC. This might be due to the widespread deployment of these varieties (with a limited number of resistance genes) in South East Asia [7,10]. In many cases, the planthoppers were virulent against the IR lines, but had reduced fitness on related putative donor varieties, suggesting either that the target genes had not been introgressed into the IR lines as expected, or that differences between the genetic backgrounds of the modern and traditional varieties influenced the strength of resistance [7,13].

The genetic similarity of many of the IR varieties (bootstrap value of 67%), often with overlapping pedigrees (i.e., with shared ancestors) [10], may reduce the degree of resistance gained from quantitative traits. However, this has apparently not been the case with resistance against *N. virescens*, as most of the IR varieties have maintained high resistance to this species (Figure 4). Although few studies have examined virulence adaptation in leafhoppers, evidence suggests that the leafhoppers can rapidly gain partial virulence (i.e., rapid adaptation to feed on resistant varieties, but slower adaptation to oviposit on the same varieties [36]) and that virulence may be less stable than in *N. lugens*. For example, relict colonies maintained on susceptible rice varieties over several years in Japan remain virulent to *Bph1* and *bph2* [44]; but there is no evidence of a similar phenomenon among virulent *N. virescens* [45].

The features of virulence adaptation revealed in this study suggest that caution must be exercised during the development and deployment of resistant rice varieties. Pyramiding resistance genes has been shown to increase the strength and durability of resistance against plant- and leafhoppers [14,15,44]; however, plant- and leafhopper populations virulent against pyramided lines have already been noted [17,44,46] and further virulence may develop in the future. Combining genes in pyramided lines for deployment in virulence hotspots such as the Punjab and Vietnam requires particular caution. For example, combining any of the genes that are currently ineffective in these regions together into pyramided lines will likely not significantly improve resistance strength or durability [17], and combining an ineffective gene together with a second, apparently effective gene may not bestow any durability advantages to the line compared to monogenic lines with the single effective gene [47]. Breeders might select resistance genes for pyramiding combinations based on the lineages of the gene donors. For example, combining genes from donors in cluster C2b-4 together with those from C2b-1 might increase the durability of the rice lines (for example, compared to using genes from donors in Cb2-1 only) as virulent individuals capable of overcoming genes from different lineages are likely to be naturally rare. Similarly, combining genes introgressed from wild rice species together with those from traditional varieties might improve durability; however, resistance genes from wild rice have been shown to be ineffective against certain planthopper populations, despite never having been deployed in farmers’ fields. These recommendations assume that virulence to major genes is determined by gene-for-gene mechanisms [48,49]. However, if planthoppers adapt to defense mechanisms (irrespective of underlying genetics) then pyramiding arbitrary gene combinations might play little role in determining durability. In such a case, increasing the number of resistance genes or combining different resistance mechanisms in a pyramided line might be more effective [25]; however, there are also limits to the number of genes that can be pyramided without corresponding ecological costs to yields or other essential plant traits [36]. Without clear knowledge of the mechanisms of plant-and leafhopper adaptation to resistance genes, it will be difficult to predict the optimal combinations of defense genes for specific geographical regions.

### 4.2 Virulence Adaptation among Sogatella furcifera in East Asia

The present study also aimed to assess the current utility of resistance genes against *S. furcifera* in Taiwan and the Philippines using the same 39 varieties tested against *N. lugens* by Horgan et al. [11]. These varieties are known to contain over 11 genes for resistance against *S. furcifera* (85% of all known *S. furcifera* resistance genes at the time of study). Myint et al. [44] had previously examined the virulence of relict *S. furcifera* populations collected in 1989, 1999 and 2005 on seven resistant varieties with seven resistance genes and found that N22 (*Wbph1*) and Mudgo (*WbphM1* and *WbphM2*) were already ineffective by 1989 and that ARC 10239 (*Wbph2*) was ineffective by 2005. At the time of that study, ADR52 (*Wbph3*), Podiwi-A (*wbph4*) and N’Diang Marie (*Wbph5*) were still effective against *S. furcifera* in Japan [44].

We confirmed that N22 is largely ineffective against *S. furcifera* in Taiwan, but not in the Philippines, whereas Mudgo was ineffective against both *S. furcifera* colonies. However, ARC 10239 showed strong resistance against the Taiwanese colony and moderate resistance against the Philippine *S. furcifera* colony. Furthermore, many of the varieties that we used that had been previously evaluated in the 1980s for their resistance against *S. furcifera* (without identifying any specific *S. furcifera* resistance genes), maintained a moderate to high level of resistance against our test populations: for example, Babawee, Balamawee, Rathu Heenati and PTB33 [34] as well as IR60 and IR62 [50] were still moderately resistant to the CAES and IRRI *S. furcifera* colonies. This was most evident in the MSST results from our study. Resistance in N22 is apparently unstable according to previous studies [51,52] and in contrast to the study by Myint et al. [44] was among the least damaged varieties in the SSSTs conducted at IRRI in the present study. Overall, our results suggest that adaptation by *S. furcifera* populations has not been as prominent as in the case of *N. lugens*. Nevertheless, screening results did suggest that the IRRI *S. furcifera* population was more virulent than the Taiwanese population to many of the varieties we tested.

### 4.3 Responses by Planthoppers to Resistant Rice Varieties and Interpretation of Seedbox Tests

A number of studies have evaluated planthopper responses to resistant varieties using no-choice tests. Many have been conducted using varieties from the present study (i.e., ADR52, ARC 10239, N22 and PTB33). These studies, have shown planthopper nymphs to have reduced settling rates, ingest and assimilate less food, and have lower survival and slower growth and development rates relative to nymphs reared on TN1 (*N. lugens*: [17,43,53]; *S. furcifera*: [33,51,54]). Furthermore, adult planthoppers have been shown to have a shorter lifespan and lay fewer eggs on resistant varieties, and their eggs often have lower hatching rates (*N. lugens*: [30]; *S. furcifera*: [33]). These combined effects reduce population growth rates and damage to the host plants [51,52]. Responses by planthoppers on wild rice species have been generally similar (*N. lugens*: [22]; *S. furcifera*: [55–58]). Furthermore, detailed analyses of feeding responses to resistant varieties indicate that planthoppers probe more and suck less on resistant plants than on susceptible rice varieties (*N. lugens*: [7,53,59]; *S. furcifera*: [34]) suggesting that some of the resistance is due to feeding inhibitors. Responses by *N. lugens* and *S. furcifera* on resistant rice plants, suggest that the underlying resistance mechanisms against both insects are similar.

The results from our fitness bioassays also indicate that planthoppers had reduced growth and laid fewer eggs on the most resistant varieties. This resulted in lower rates of population build-up in bioassays with older plants. The results from the fitness tests conducted at IRRI generally correlated well with results from the seedbox tests. Analyses of honeydew excretion conducted at DRR was poorly correlated with the results from the corresponding SSSTs at that center, but better correlated with the MSSTs. Furthermore, the honeydew tests clearly differentiated Triveni, with apparent tolerance to planthoppers, from the remaining resistant varieties. High levels of honeydew production on the tolerant variety, but relatively low damage scores suggest that the planthoppers successfully fed on the variety without causing excessive damage. A further tolerant variety, Utri Rajapan [25], also appears to be moderately resistant to *N. lugens*.

Knowledge of the resistance mechanisms of rice against *S. furcifera* or *N. lugens* has increased in recent years, albeit with much of the information related to only a small number of resistance genes [7,25,60]. Resistance associated with the *Bph14* gene is related to an immune receptor of the NB-LRR family which activates salicyclic acid and jasmonate mediated defense pathways [60]. Similarly, the ovicidal response [7,25,61] is an induced response involving the formation of lesions, with necrosis of parenchymal cells around the point of egg insertion into the rice plant. The lesions become fully or partially filled with benzyl benzoate that eventually kills the eggs. Egg mortality due to the ovicidal response is normally higher in *S. furcifera* than in *N. lugens*; furthermore, the ovicidal response, which is largely governed by the *Ovc* gene as well as several QTLs, occurs predominantly in older plants [30,61]. Strong ontogenic effects on the ovicidal response against *S. furcifera* were apparent in the present study as relatively strong resistance of Asiminori in the MSSTs, but high susceptibility in the SSSTs. [30,61]. The apparently strong reaction by planthoppers to Asiminori in the present study indicates the value of the ovicidal response for rice resistance against *S. furcifera* and the utility of employing additional screening tests such as the MSST during rice breeding programs.

### 4.4 Origins of Resistance and Local Adaptation

A prominent paradigm underlying modern resistance breeding is that genes bestow resistance against herbivores in a gene-for-gene mechanism [48,49]. This paradigm has gained some recent support through the identification of a ‘virulence gene’ associated with planthopper adaptation to *Bph1* [49]. However, a study conducted at about the same time and using a different *N. lugens* population virulent to the same *Bph1* gene, identified a different ‘virulence gene’ and associated ‘virulence QTLs’ [48]. Furthermore, previous studies have indicated the role of regional adaptation by planthoppers to locally popular rice varieties: for example, Claridge and Den Hollander [19] have shown that *N. lugens* from Australia had poor survival on the highly susceptible Asian variety TN1. Furthermore, Claridge et al. [20] indicated that *N. lugens* populations from Sri Lanka had highest fitness when reared on varieties from the regions where each population was originally collected, but had low fitness on varieties from other regions. These observations might suggest that the high resistance of Indian and Sri Lankan varieties in cluster C2b-1 is due to these varieties representing unfamiliar hosts for Philippine *N. lugens* populations; however, resistance against Indian *N. lugens* was also often high among these varieties. We examined the relation between genetic distance from TN1 and resistance scores for *N. lugens, S. furcifera* and *N. virescens* in the present study, and although resistance was often slightly higher among the most divergent varieties within each cluster, there were few significant correlations. The materials used in this study were therefore mainly resistant because of their major genes with two noted regions of high planthopper virulence, one in Punjab and one in Vietnam, suggesting a high degree of selection pressure either from widespread planting of traditional resistant varieties in the historical past (pre-Green Revolution Punjab) or because of the extensive use of a limited number of resistance donors in modern breeding programs (Vietnam).

### 4.5 Research Center Origins of Resistance against Nephotettix virescens

Whereas resistance in cluster C2b-1 has a likely phylogenetic origin geographically linked to India and Sri Lanka, the apparent high resistance of IR varieties against *N. virescens* and the resistance in IR56, IR62 and IR65482-4-136-2-2 against both *N. virescens* and *N. lugens* has an apparent research center origin. That is, the high resistance noted among these varieties has been due to effective rice breeding activities. These IR varieties were selected for this study because of their resistance to *N. lugens* without any prior knowledge of their resistance to *N. virescens*. Among the varieties, only IR56 and IR60 are known to contain genes for resistance to *N. virescens* (*Glh9* gene: [62]). *Nephotettix virescens* is an important pest in South East Asia largely because it is a vector of tungro virus [7,45]. IRRI’s breeding program has traditionally included routine screening of all materials for resistance to *N. lugens* and *N. virescens*. This screening has relied heavily on SSST phenotyping in greenhouses using local *N. lugens* and *N. virescens* colonies [5] with a similar geographic origin to the colonies used at IRRI in the present study.

Although SSST screening often fails to differentiate between the relative strengths of resistant varieties or breeding lines because it is evaluated against the highly susceptible TN1, it can be used to successfully exclude highly susceptible materials from breeding programs [5]. Furthermore, the elimination of rice lines because of their susceptibility to tungro disease during breeding programs, which is apparent as obvious yellowing in field-grown plants [7], would have helped maintain the low susceptibility of IRRI’s lines to *N. virescens* [63]. Although, lines are also screened for resistance to *N. lugens* during IRRIs breeding program, any lines that pass through initial seedbox screening to become advanced breeding lines would not have been further evaluated in the field, since planthopper incidences are normally low under proper field management and ‘hopperburn’ has been successively declining at IRRI over recent decades [64]. In contrast, small numbers of leafhoppers can transmit tungro virus, which is generally apparent in infected rice as yellowing and stunting of tillers. This suggests that field activities may have further improved the resistance of IR varieties against *N. virescens*, but not against *N. lugens*, and that this resistance has mainly been due to quantitative traits. High resistance against *N. lugens* in some of the varieties from C2b-2 is apparently due to the introgression of known resistance genes from wild rice species using marker assisted selection (as in the case of IR71033-121-15 and IR65482-4-136-2-2).

Our results therefore indicate the importance of combining phenotyping and genotyping for resistance to successfully develop field resistant rice varieties. Reduced funding for breeding programs, reduced attention to phenotyping, and marker assisted selection without phenotypic evaluation of resulting lines [5] are all likely to result in a greater susceptibility of field materials. Breeding programs should therefore continue to conduct screening of materials using phenotyping tests and at the same time include resistance donors from diverse clades in their breeding pedigrees. Further research is also necessary to ensure that varieties are carefully deployed to avoid the selection of virulent planthoppers and leafhoppers.

## Supplementary Materials

The following are available online at http://www.mdpi.com/2073-4395/7/4/62/s1, Table S1: Rice varieties used in the present study with putative resistant genes and QTLs. Table S2: *p*-values for Dunnett’s ‘many to one’ comparisons between *Nilaparvata lugens* damage scores for varieties tested at seven research centers against the susceptible variety TN1.
