# Supplementary material for: Geographic and Research Center Origins of Rice Resistance to Asian Planthoppers and Leafhoppers: Implications for Rice Breeding and Gene Deployment
Source: Agronomy (Basel). 2017 Sep 21;7:62. doi: 10.3390/agronomy7040062 (PMC7371011; doi:10.3390/agronomy7040062)
Supplement: Supplementary file 1 [file A-2017-AGRONOMY7040062-s1.docx]

**Table S1.** Rice varieties used in the present study with putative resistant genes and QTLs. See Horgan et al [11] for further details.

| **Variety (accession)** | **Origin** | **Identified resistant genes** | **Variety deployment** |
| --- | --- | --- | --- |
|  |  |  |  |
| ADR52 (Ac. 40638) | India | *BPH25, BPH26, Wbph3* | Landrace/traditional cultivar |
| ARC 10239 (IRTP 9677) | India | *Wbph2* |  |
| ARC 10550 (Ac. 12507) | India | *bph5* |  |
| ARC 6650 (Ac. 12308) | India | *wbph4* (resistant to *N. lugens* in South India) |  |
| ASD7 (Ac. 6303) | India | *bph2, Glh2* | Released advanced cultivar |
| Asiminori (Ac. 39942) | South Korea | ovicidal response to *S. furcifera* | Released/advanced cultivar |
| Babawee (Ac. 6730) | Sri Lanka | *bph4* | Landrace/traditional cultivar |
| Balamawee (Ac. 8919) | Sri Lanka | *Bph9,Bph27(t)* | Landrace/traditional cultivar |
| Chinsaba (Ac. 33016) | Myanmar | *bph8* | Breeding/inbred line |
| Da Hua Gu (Ac. 79597) | China | *Wbph6* |  |
| IR22 (Ac. 11356) | IRRI | None | IRS/Tadukan, released in 1969 |
| IR24 | IRRI | *Bph1,Grh*1 | IR8/IR127-2-2 released in 1971 |
| IR40 | IRRI | *bph2* | Peta//Peta*3/TN1, released in 1977 |
| IR56 | IRRI | *Bph3 , Glh9* | IR4432-53-33/PTB33//IR36, released in 1982 |
| IR60 | IRRI | *Bph32* | Released in 1983 |
| IR62 | IRRI | *Bph32* | PTB33/IR30//IR36, released in 1984 |
| IR64 | IRRI | *Bph1* and unidentified QTLs | lR5657-33-2-1/lR2061-465-1-5-5, released in 1985 |
| IR65482-4-136-2-2 (IRTP 19029) | IRRI | *Bph10* | IRRI breeder line developed though introgression with *Oryza australiensis* Domin |
| IR65482-7-216-1-2 (IRTP 26968) | IRRI | *Bph18* | IRRI breeder line developed though introgression with *O. australiensis* |
| IR66 | IRRI | *bph4* | lR13240-108-2-2-3/IR9129-209-2-2-2-1, released in 1987 |
| IR70 | IRRI | *Bph32* | lR19660-73-4/lR54//IR9828-36-3, released in 1989 |
| IR71033-121-15 (IRTP 23991) | IRRI | *Bph*20(t), *Bph*21(t), *Bph*23(t), *qBPH6*(t) | IRRI breeder line developed though introgression with *Oryza minuta* JS Presl ex CB Presl |
| IR72 | IRRI | *Bph3* | R19661-9-2-3/IR15795-199-3-3//IR9129- 209-2-2-2-1, released in 1990 |
| IR74 | IRRI | *Bph3* | lR19661-131-1-2/IR15795-199-3-3, released in 1991 |
| Mudgo (Ac. 6663) | India | *Bph1, WbphM1, WbphM2* | Released advanced cultivar |
| Nagina 22(N22: Ac. 6264) | India | *Wbph1* |  |
| N'Diang Marie (Ac. 15859) | Senegal | *Wbph5* |  |
| Pokkali (Ac. 15602) | Sri Lanka | *Bph9* | Landrace/traditional cultivar |
| PTB33 (Ac. 19325) | India | *bph2, Bph3* (*BPH32* [16]), *Zlh3* | Released advanced cultivar |
| Rathu Heenati (Ac. 11730) | Sri Lanka | *Bph3, Bph17, Zlh1* | Landrace/traditional cultivar |
| Swarnalata (Ac. 33964) | Bangladesh | *Bph6,Qbph8* | Breeding/inbred line |
| T65 (Ac. 79/IRTP 07579) | Taiwan | None |  |
| Taichung Native 1 (TN1) | Taiwan | None | Released/advanced cultivar in 1960 |
| Triveni (Ac. 14785) | India | Tolerance | Released/advanced cultivar in 1973 |
| Utri Rajapan (Ac. 16684) | Indonesia | Tolerance to *N. lugens* and some resistance to *S. furcifera* | Landrace/traditional cultivar |
| Yagyaw (Ac. 33849) | Vietnam | *4 QTLs - Qbph3, Qbph9* | Breeding/inbred line |
