# Supplementary material for: Geographic and Research Center Origins of Rice Resistance to Asian Planthoppers and Leafhoppers: Implications for Rice Breeding and Gene Deployment
Source: Agronomy (Basel). 2017 Sep 21;7:62. doi: 10.3390/agronomy7040062 (PMC7371011; doi:10.3390/agronomy7040062)
Supplement: Supplementary file 2 [file A-2017-AGRONOMY7040062-s2.docx]

**Table S2.** P-values for Dunnett’s ‘many to one’ comparisons between *Nilaparvata lugens* damage scores for varieties tested at seven research centers against the susceptible variety TN1 (see Figure 2)

| **Variety** | **IRRI (Philippines)** | | **SRPPC (Vietnam)** | | **CAES (Taiwan)** | | **DRR**  **(India)** | | **Pioneer**  **(India)** | | **APRRI**  **(India)** | | **PAU**  **(India)** | |
| --- | --- | --- | --- | --- | --- | --- | --- | --- | --- | --- | --- | --- | --- | --- |
|  | **SSST** | **MSST** | **SSST** | **MSST** | **SSST** | **MSST** | **SSST** | **MSST** | **SSST** | **MSST** | **SSST** | **MSST** | **SSST** | **MSST** |
| ARC 10239 | 1.000 | 0.181 | 1.000 | 0.001 | 1.000 | 0.277 | 1.000 | 0.743 | 1.000 | 1.000 | - | - | 1.000 | 0.752 |
| N22 | 1.000 | 0.573 | 1.000 | 0.750 | 1.000 | 1.000 | 0.168 | 0.998 | 1.000 | 1.000 | 0.767 | 0.767 | 1.000 | 0.436 |
| Asiminori | 0.314 | **0.023** | - | - | - | - | 1.000 | - | - | - | - | - | 0.961 | 0.525 |
| ADR52 | 0.638 | **0.023** | 0.326 | **<0.001** | **<0.001** | **<0.001** | **0.001** | **<0.001** | **<0.001** | **<0.001** | **<0.001** | **<0.001** | **<0.001** | **<0.001** |
| ARC 6650 | **0.002** | 1.000 | 0.992 | **<0.001** | **0.003** | **<0.001** | 0.121 | **<0.001** | 0.378 | 0.922 | **<0.001** | **<0.001** | 0.904 | 0.446 |
| Balamawee | **<0.001** | 0.252 | 0.326 | **0.014** | **<0.001** | **<0.001** | 0.903 | **<0.001** | **<0.001** | **<0.001** | **<0.001** | **<0.001** | 1.000 | 0.348 |
| Ptb33 | **<0.001** | **0.023** | **<0.001** | **0.014** | **<0.001** | **0.005** | **0.001** | **<0.001** | **<0.001** | **<0.001** | **<0.001** | **<0.001** | **<0.001** | **<0.001** |
| Rathu Heenati | **<0.001** | **0.023** | **0.010** | 0.103 | **<0.001** | **<0.001** | 0.520 | **<0.001** | **<0.001** | **<0.001** | **<0.001** | **<0.001** | **<0.001** | **<0.001** |
| IR56 | **<0.001** | 0.252 | 0.740 | **0.040** | - | - | - | - | - | - | - | - | - | - |
| IR62 | **<0.001** | 0.252 | 0.740 | **0.040** | **<0.001** | **<0.001** | 0.903 | **<0.001** | **0.017** | 0.024 | 0.939 | 0.939 | **<0.001** | **<0.001** |
| IR65482-4-136-2-2 | **<0.001** | 0.252 | 1.000 | **0.004** | 0.137 | **<0.001** | 1.000 | **<0.001** | 0.161 | 0.256 | **0.016** | **0.016** | **<0.001** | **<0.001** |
| IR22 | 1.000 | 1.000 | 1.000 | 1.000 | 1.000 | 1.000 | 1.000 | 0.152 | 0.097 | 1.000 | **0.047** | **0.047** | 1.000 | 0.261 |
| IR24 | **0.039** | 0.995 | 1.000 | 0.750 | 0.945 | 1.000 | 1.000 | **<0.001** | 0.378 | 0.256 | **<0.001** | **0.001** | 1.000 | 0.162 |
| IR40 | 0.938 | 0.973 | 1.000 | 0.967 | 1.000 | 1.000 | 1.000 | 0.167 | 0.961 | 1.000 | 0.998 | 0.998 | 0.368 | 0.572 |
| IR66 | **0.016** | 1.000 | 0.992 | 0.103 | **<0.001** | **<0.001** | 0.750 | **<0.001** | 0.378 | 0.581 | 0.334 | 0.334 | 1.000 | 0.648 |
| IR70 | 0.862 | 1.000 | 0.740 | 0.750 | 0.137 | 0.046 | 1.000 | **<0.001** | 0.708 | 0.581 | - | - | 0.993 | **0.005** |
| IR71033-121-15 | 0.085 | 0.086 | 0.326 | **0.002** | **0.003** | **<0.001** | - | - | **<0.001** | **<0.001** | **<0.001** | **<0.001** | **<0.001** | **<0.001** |
| IR72 | 1.000 | 0.181 | 0.992 | 0.455 | **0.025** | **<0.001** | 1.000 | **<0.001** | 0.708 | 0.922 | **0.002** | **0.002** | **<0.001** | **<0.001** |
| IR74 | **0.026** | 0.086 | 0.326 | **0.014** | **<0.001** | **0.005** | 0.998 | **<0.001** | **0.004** | **<0.001** | - | - | **<0.001** | **<0.001** |
| Swarnalata | 0.171 | 0.086 | **0.023** | 0.233 | **<0.001** | **0.005** | 0.566 | **<0.001** | **0.056** | 0.087 | **0.006** | **0.006** | 0.971 | - |
| Yagyaw | 1.000 | 0.998 | 1.000 | **0.004** | 0.945 | 0.830 | 0.177 | **<0.001** | 0.056 | 0.256 | **<0.001** | **<0.001** | 1.000 | 0.098 |
| IR64 | **0.006** | 0.702 | 0.326 | **0.004** | 0.137 | **0.046** | 1.000 | - | 0.161 | 0.564 | 1.000 | 1.000 | - | - |
| IR65482-7-216-1-2 | 0.519 | 1.000 | 1.000 | 0.103 | 1.000 | 1.000 | 0.969 | **<0.001** | 0.378 | 1.000 | - | - | - | - |
| IR60 | 1.000 | 1.000 | 0.740 | 0.103 | **<0.001** | **<0.001** | - | - | 0.708 | - | - | - | - | - |
| T65 | 1.000 | 0.252 | 1.000 | 1.000 | - | - | - | - | 1.000 | - | - | - | - | - |
| ASD7 | 0.235 | 0.573 | 1.000 | 0.455 | **0.025*** | **0.005** | 0.705 | 0.291 | 0.708 | 1.000 | **<0.001** | **<0.001** | 1.000 | 0.593 |
| Pokkali | **<0.001** | 0.973 | 0.992 | 0.103 | 0.488 | **0.005** | 0.498 | **<0.001** | 0.961 | 0.087 | - | - | 1.000 | 0.156 |
| ARC 11367 | - | - | - | - | 0.488 | 1.000 | 1.000 | **<0.001** | **0.004** | **0.024** | **0.016** | **0.016** | 1.000 | 0.529 |
| Jai Nong 66 | - | 0.450 | - | - | - | - | 1.000 | - | 1.000 | - | - | - | 1.000 | 0.596 |
| MO1 | - | - | 0.740 | 0.455 | **<0.001** | **0.005** | **<0.001** | **<0.001** | **0.017** | **0.005** | **<0.001** | **<0.001** | **<0.001** | **<0.001** |
| ARC 10550 | **0.010** | 1.000 | 1.000 | **<0.001** | 1.000 | 1.000 | 0.338 | **0.032** | 0.708 | **0.024** | **0.005** | **0.005** | **<0.001** | **<0.001** |
| Babawee | **<0.001** | **0.023** | 0.992 | **<0.001** | **<0.001** | **<0.001** | 0.913 | **<0.001** | 0.161 | **0.005** | **<0.001** | **<0.001** | **<0.001** | 0.088 |
| Chinsaba | 0.862 | **0.023** | 1.000 | **0.014** | 1.000 | 1.000 | 1.000 | **<0.001** | 0.378 | 0.581 | **0.003** | **0.003** | 1.000 | 0.873 |
| Da Hua Gu | 1.000 | 0.973 | 1.000 | 1.000 | 0.945 | 1.000 | 1.000 | 0.106 | 0.161 | 1.000 | 0.939 | 0.939 | 0.716 | 0.436 |
| Mudgo | 0.757 | 0.086 | 0.740 | **<0.001** | **0.025** | **0.046** | 0.272 | **<0.001** | 0.056 | 0.256 | **<0.001** | **<0.001** | **<0.001** | **<0.001** |
| N'Diang Marie | 1.000 | 0.573 | 0.326 | 0.103 | 0.025 | 0.005 | 1.000 | **<0.001** | 0.961 | 1.000 | **<0.001** | **<0.001** | 0.055 | 0.384 |
| Triveni | 0.085 | 0.702 | 1.000 | 0.103 | **0.003** | **<0.001** | 0.998 | 0.852 | 1.000 | 1.000 | **0.004** | **0.004** | 1.000 | 0.843 |
| Utri Rajapan | 0.862 | 0.252 | 1.000 | 0.040 | 0.488 | 0.277 | 0.158 | 0.001 | 0.161 | 0.256 | 0.087 | 0.087 | 0.993 | 0.030 |
| F-variety^1^ | 6.656 | 2.617 | 8.386 | 4.342 | 7.193 | 9.080 | 5.759 | 25.926 | 4.195 | 6.917 | 10.727 | 10.949 | 35.061 | 10.668 |
| DF | 35 | 36 | 35 | 35 | 34 | 34 | 34 | 31 | 36 | 33 | 28 | 28 | 33 | 32 |
| Error | 72 | 74 | 72 | 71 | 70 | 70 | 68 | 47 | 72 | 67 | 58 | 58 | 68 | 66 |
| Spearman Rho | 0.234 |  | 0.355 |  | 0.838 |  | 0.559 |  | 0.847 |  | 0.615 |  | 0.611 |  |
| P-values | 0.170 |  | **0.033** |  | **<0.001** |  | **0.001** |  | **<0.001** | | **<0.001** |  | **<0.001** |  |

1: All associated P-value were < 0.001
